# Dilatometric Study of Phase Transformations in 5 Mn Steel Subjected to Different Heat Treatments

**DOI:** 10.3390/ma13040958

**Published:** 2020-02-21

**Authors:** Mateusz Morawiec, Adam Grajcar, Władysław Zalecki, Carlos Garcia-Mateo, Marek Opiela

**Affiliations:** 1 Department of Engineering Materials and Biomaterials, Silesian University of Technology, 18A Konarskiego Street, 44-100 Gliwice, Poland; Adam.Grajcar@polsl.pl (A.G.); marek.opiela@polsl.pl (M.O.); 2 Łukasiewicz Research Network—Institute for Ferrous Metallurgy, 12-14 K. Miarki Street, 44-100 Gliwice, Poland; Wladyslaw.Zalecki@imz.pl; 3 National Center for Metallurgical Research, Av. de Gregorio del Amo, 8, 28040 Madrid, Spain; cgm@cenim.csic.es

**Keywords:** medium manganese steel, heat treatment, dilatometric study, TRIP steel, retained austenite

## Abstract

The work presents results of phase transformation kinetics of hot-rolled 5% Mn steel subjected to different heat treatments. Three different schedules were introduced: isothermal holding in a bainite region, coiling simulation and intercritical annealing. The evolution of microstructure components was investigated using dilatometric and metallographic analyses. According to obtained results, the medium-Mn steel exhibits high resistance for γ/α transformation during the bainite transformation and coiling simulation (upon cooling from the austenite region). During 5 h isothermal holding, no bainite and/or ferrite formation was detected. This results in the formation of martensite upon cooling to room temperature. Differently, when the steel was subjected to the intercritical annealing at 720 and 700 °C (upon heating from room temperature), a final microstructure consisted of ferrite, martensite and retained austenite. At 700 °C, no fresh martensite formation was detected upon cooling to room temperature. This means that the austenite was enriched in carbon during the intercritical annealing step enough to keep its thermal stability.

## 1. Introduction

Medium manganese steels are some of the most promising grades of steel in the automotive industry. The reason for this is a good compromise between material cost and mechanical properties. The metastable retained austenite, one of the main phases, is responsible for high combinations of strength and ductility [1,2]. This phase during deformation transforms locally into martensite increasing both strength and ductility of the steel. This prevents the strain localization increasing the deformation potential of steel elements. That is why high manganese austenitic steels have very promising mechanical properties [3]. The problem is the price of such steels, which limits their application in car body construction. Therefore, the medium manganese steels have recently become of particular scientific interest [4,5,6]. 

The utilization of the beneficial microstructure–mechanical properties relationships is only possible when the knowledge on phase transformation kinetics required for designing the heat treatment schedules is available. Increased Mn and Al additions are new in structural steels. Therefore, there is a need to develop phase transformations diagrams for steels containing new ranges of alloying elements. This should be done combining computational and experimental approaches [1].

The presence of retained austenite is due to its high thermal stability revealing during heat treatment. Its stability is influenced by carbon and manganese contents in the austenite, a morphology, dislocation density and the neighborhood of soft or hard phases [7,8,9]. These factors determine the amount of retained austenite present in the microstructure and resulting mechanical properties. A good way to determine the stability of austenite is dilatometry. This method allows for analyzing changes in a sample length during heat treatment. The change in length corresponds to the phase transformation undergoing in the material. There are possibilities to determine critical temperatures of steel: A_c1_, A_c3_, martensite start (M_s_) and finish (M_f_) temperatures. The rate of transformation can be also analyzed.

The reasons why austenite remains in the microstructure are different for hot-rolled steel sheets and cold-rolled steel sheets. Thus, once hot rolling in the austenitic field has been applied, either coiling [8] or austempering [1,2] is usually applied so austenite is retained in the final microstructure as part of its incomplete transformation on final cooling. On the contrary, the cold-rolled medium-Mn steels require intercritical annealing as a final heat treatment step [6,7]. In such cases, the austenite is formed upon heating conditions. These three possible heat treatment routes are simulated in the present work. 

## 2. Material and Experiments 

### 2.1. Material

A laboratory-melted medium manganese steel was studied in this work and its chemical composition is detailed in Table 1. A relatively low carbon content of the investigated steel (0.16 wt.%) is beneficial due to the improvement in steel weldability [10]. The 4.7 wt.% of manganese was added to enhance the thermal stability of austenite upon cooling. A high aluminum content (1.6 wt.%) prevents the carbide precipitation during a bainite reaction, hence increasing the carbon content in the austenite [11]. Moreover, aluminum increases the rate of γ to α transformation (increases the chemical driving force) [12,13]. Silicon has a similar role as aluminum, yet its content was limited due to its disadvantages during hot-dip galvanizing. This element forms a thin amorphous layer which prevents the good diffusion connection between steel and zinc [2]. 

The initial microstructure of the steel before heat treatment was martensite (formed during air cooling after hot forging). This is the result of the high hardenability of the analyzed steel.

### 2.2. Experimental Details

**Figure 1:**
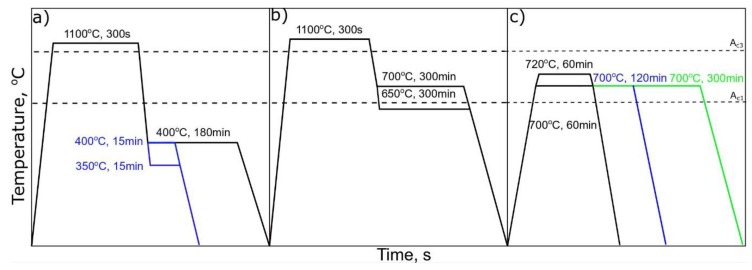
Different heat treatment schedules for analyzed steel: (**a**) in bainite region; (**b**) coiling simulation (ferrite region); (**c**) intercritical annealing.

The aim of the study was to determine the effect of different heat treatments on the formation and stability of retained austenite in the steel containing 5% Mn and the increased Al addition. In order to minimize the number of tests and also avoid a trial-error approach, in the first step of the research, theoretical calculations based on the above chemical composition were carried out by the JMatPro (database ver. 11.2) general steel module [14]. The MUCG (database ver. 83) software was applied to calculate the effects of manganese and aluminum on the Gibbs free energy changes in the case of the analyzed steel [15]. The calculations in equilibrium and non-equilibrium conditions were made to determine appropriate heat treatment schedules for the analyzed steel. Based on the calculations, the parameters for the three different treatments were selected. The isothermal holding in a bainite region and the coiling simulation routes reflected steel cooling from the austenitization step, whereas the third one, intercritical annealing, corresponded to the microstructure formation upon heating from room temperature (see Figure 1 for a general scheme of the described routes). Note that the treatment parameters shown in Figure 1 are the result of theoretical calculations, as it will become clear in the following sections of the manuscript. The isothermal treatment in Figure 1a (or variant A) was performed in the bainitic region of the steel. This approach allows for the austenite stabilization in a process of carbon rejection from bainitic plates. The second treatment or variant B (Figure 1b) corresponds to the coiling simulation. In this case, the presumable formation of ferrite is predicted. Ferrite similarly to bainite rejects carbon, which diffuses to austenite increasing its thermal stability. The last performed treatment (Figure 1c) was an intercritical annealing, variant C. The formation of ferrite during heating and annealing steps increases the carbon content in the austenite. The distinguishable feature between the three described treatments is the microstructure right before isothermal holding. Thus, in the case of the variants A and B, the microstructure is 100% austenite, while for variant C, which exploits reverse austenite transformation, the initial microstructure is composed of ferrite and austenite.

The experimental procedure was carried out by the means of dilatometry using a high-resolution BAHR Dilatometer DIL805A/D. The analysis and determination of transformation temperatures were performed according to the ASTM A1033-04 standard [16]. Dilatometric samples of 4 mm diameter and 10 mm length were used in all experiments. The heating of samples was performed by an induction system in a vacuum and the temperature controlled by a K type thermocouple welded to the center of the specimen. The samples were heated up at the rate of 3 °C/s to a temperature of 1100 °C (variants A and B). The austenitization time was 300 s, after which the samples were cooled down at 60 °C/s, by blowing He directly into the specimens, down to selected temperatures. In the case of the variant C, the material was heated to an intercritical temperature of 700 and 720 °C with the same heating rate. After the isothermal holding, samples in all variants were cooled down to room temperature at a rate of 10 °C/s. The longer time was designed because of manganese diffusion, which needs more time to enrich austenite. It is necessary because the manganese is an austenite forming element. Therefore, it enhances the stability of retained austenite [17].

After the dilatometry tests, the specimens were prepared for light microscopy (LM) and scanning electron microscopy (SEM) observations according to standard metallographic procedures. They were cut in the center, perpendicular to their length, mechanically ground with SiC paper up to 2000 grid, polished with a diamond paste and finally etched in 5% nital. 

## 3. Results 

### 3.1. Theoretical Calculations

**Figure 2:**
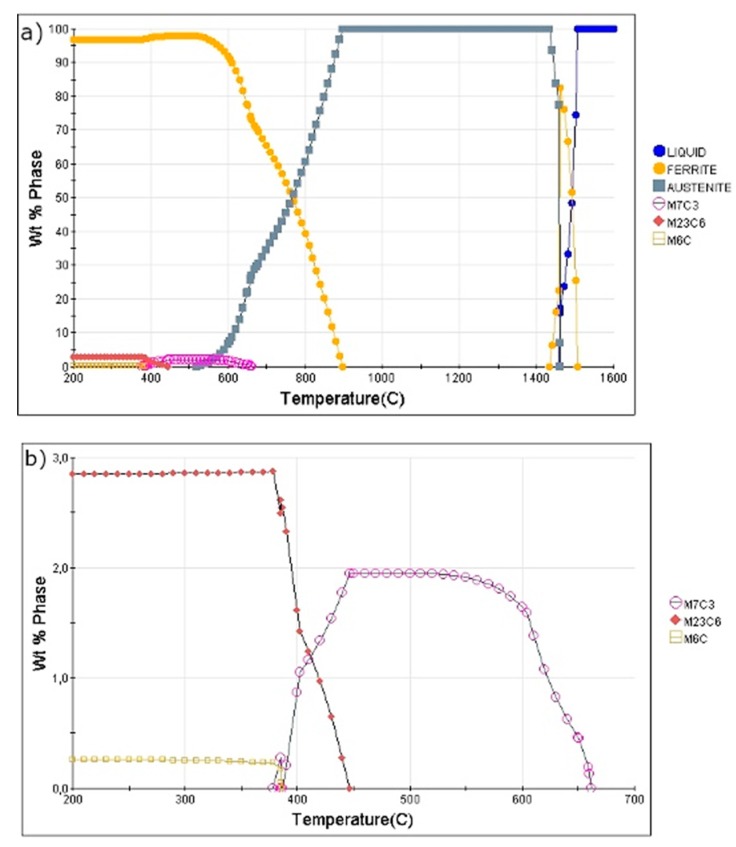
Phase evolution diagram of the steel in equilibrium conditions: (**a**) full diagram; (**b**) left bottom magnified part of the diagram.

As already mentioned, in the first approach, a set of theoretical calculations allowed the determination of the optimal parameters for the pursued treatments in this work, and for that means, JMatPro software was used. Thus, Figure 2a presents the phase evolution diagram of the analyzed steel in equilibrium conditions. According to this diagram, the γ phase is present from 900 °C up to ca. 1430 °C. Below 900 °C, the formation of ferrite takes place. This transformation occurs up to 520 °C. According to this, the A_c1_ temperature is equal to 520 °C and the A_c3_ ~900 °C (this is the intercritical region). Moreover, it can be seen that according to the equilibrium calculations, below 700 °C, the formation of some carbides can take place. These carbides may decrease the stability of austenite. Figure 2b shows that M_7_C_3_ can precipitate below 660 °C. At 450 °C, the carbides start to dissolve whereas M_23_C_6_ carbides can be formed. A very small fraction of M_6_C is also possible at the lowest temperature range. The theoretical calculations show that the austenitization temperature of 1100 °C, which is similar to industrial processing, is enough for this steel. Yet, this represents the phase evolution during cooling in equilibrium conditions.

**Figure 3:**
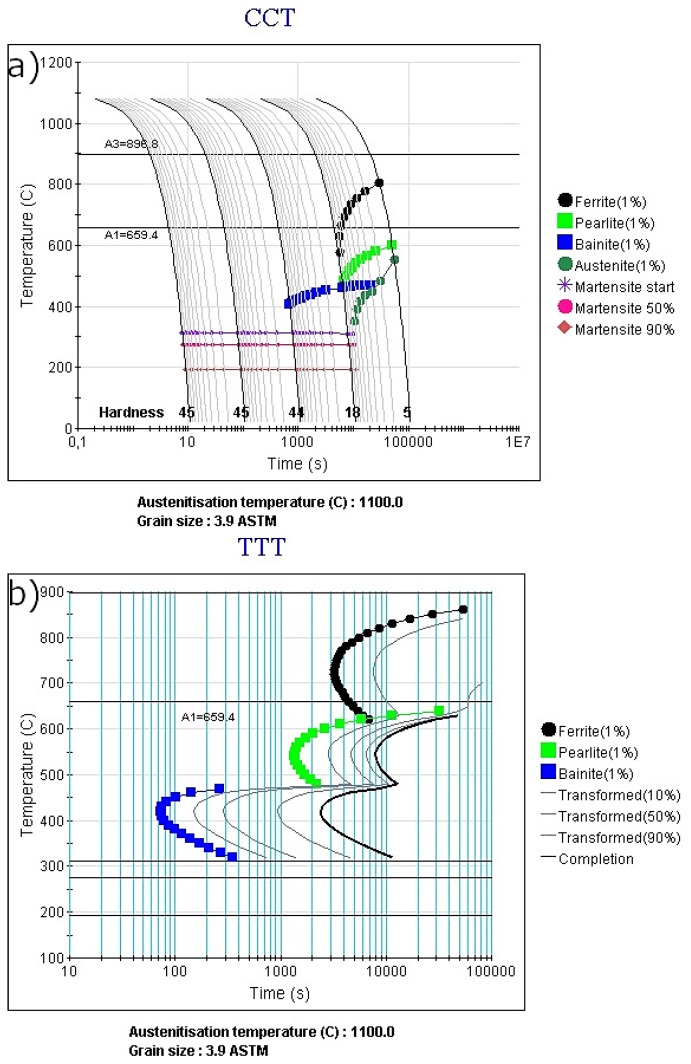
Non-equilibrium diagrams for analyzed steel: (**a**) CCT diagram; (**b**) TTT diagram.

Figure 3a,b presents the CCT (Continuous Cooling Transformation) and TTT (Temperature Time Transformation) diagrams, respectively, which are calculated for non-equilibrium cooling conditions. According to these calculations, the A_c1_ temperature is higher compared to the equilibrium conditions. During the non-equilibrium heating conditions, this temperature is equal to 660 °C (Figure 3a). This is much higher compared to 520 °C in equilibrium conditions. The reason for this is diffusion, which cannot keep up with a high heating rate [18]. Therefore, more time is necessary for it. Hence, the higher temperature of ferrite to austenite formation occurs. The starting temperature of martensite is equal to 270 °C. The bainite formation should start after 60 s at 400 °C. The transformation should be completed after 1700 s obtaining the full bainitic microstructure. At 350 °C, the transformation should start a few seconds later (150 s) and finish after 3500 s. This means that the longer time is necessary for bainite transformation to be completed at the lower temperatures.

For ferrite formation, during coiling simulation, a longer time is necessary in comparison to bainite formation. The ferrite formation at both 700 and 600 °C should start after 2000 s. According to the isothermal TTT diagram, a very long time is necessary to complete the transformation. This means that the coiling could be a good approach to stabilize some austenite during very slow cooling typical for coiling conditions. These calculations were used to determine possible heat treatments, which are presented in Figure 1.

### 3.2. Heat Treatment in Bainitic Region

The first selected heat treatment was isothermal holding in the bainite region, variant A (Figure 1a). During this treatment, the formation of carbide-free bainite should enrich austenite in carbon, rejected from bainitic plates. The presence of high Al content prevents the formation of carbides in bainite, which further causes an increase of the carbon content in the austenite. 

**Figure 4:**
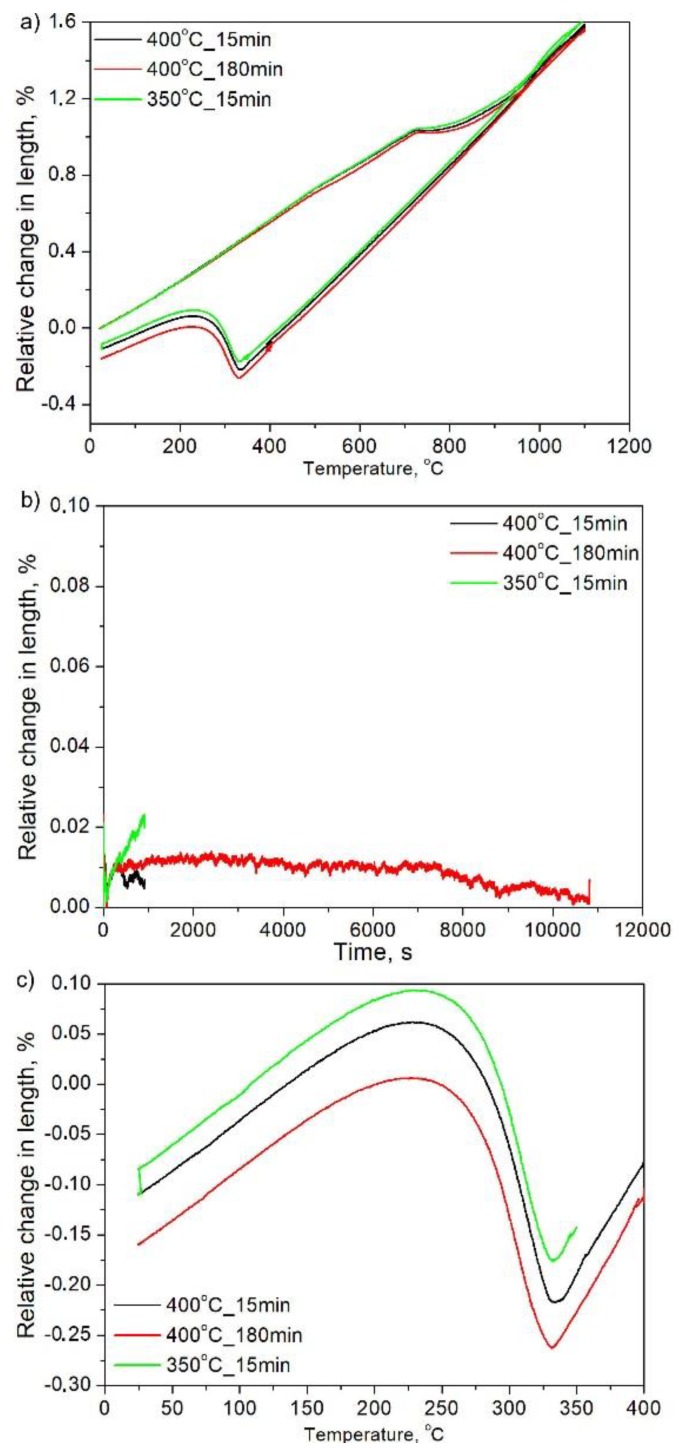
Dilatometric curves of steel during isothermal bainitic transformation: (**a**) full curves; (**b**) during isothermal holding; (**c**) during cooling to room temperature.

**Figure 5:**
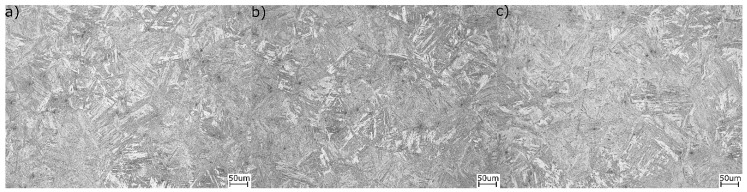
Microstructure of steel after isothermal holding in bainite region: (**a**) 400 °C, 15 min; (**b**) 400 °C, 180 min; (**c**) 350 °C, 15 min.

According to dilatometric results (Figure 4a) of this heat treatment, the A_c1_ and A_c3_ were equal to 680 and 950 °C, respectively. The level of reproducibility shown is excellent as the three shown curves almost overlap. However, during isothermal holding (see Figure 4b), regardless of the temperature, it is clear that no bainite was formed, as the relative change in length obtained is neglectable, indicative that no transformation took place for the selected isothermal times, even after 3h at 400 °C. Figure 4c presents the dilatometric curve during cooling from isothermal temperature to room temperature. The increase of relative change in length indicates the formation of fresh martensite upon cooling to room temperature. According to this, the starting temperature of martensite for all curves is 345 °C and the finish temperature is equal to 165 °C. The lack of change in the starting temperature is also a clear indicator that no transformation of austenite took place on the previous step, isothermal holding, and its chemical composition corresponds to that of the bulk for the three tested conditions, i.e., showing the same martensite starting temperature values. In summary, regardless of the chosen isothermal temperature or time, no bainitic transformation was detected during the isothermal step and only martensite forms on cooling to room temperature, as the microstructures in Figure 5 also show. Thus, for both 400 and 350 °C isothermal holding temperatures, the obtained microstructures are identical. For all the above reasons, it is clear that the applied approach did not give expected results. There was no bainite formation, thus it caused no change in the stability of austenite (no carbon enrichment). Therefore, this structural component was absent in the final microstructure.

### 3.3. Coiling Simulation Approach 

The second approach, variant B (Figure 1b), was to simulate the coiling process of the steel sheet. During industrial manufacturing, a very long time is necessary to cool down the rolled coil. This gives the opportunity to use this step to stabilize the austenite. During the manufacturing process, the coiling temperature is usually in the range of 800 to 500 °C. According to Figure 3 (theoretical calculations) during continuous cooling, the ferrite should start forming at a cooling rate of 0.1 °C/s. Hence, the intercritical region of the steel can be used to form a microstructure composed of ferrite and austenite.

**Figure 6:**
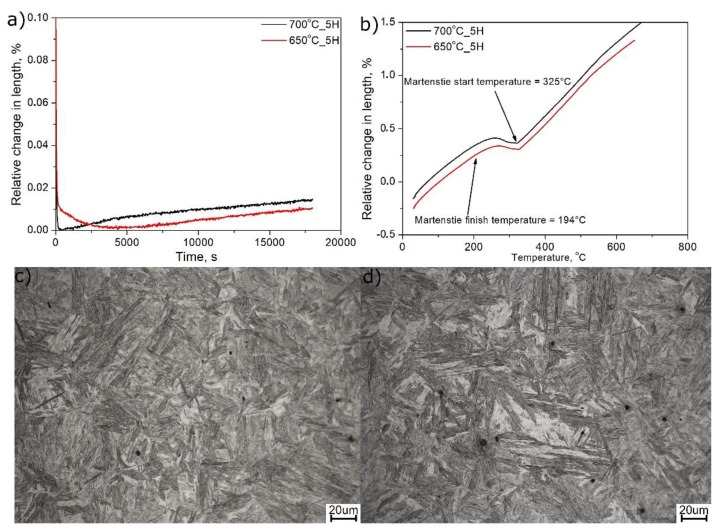
Results of dilatometry and microstructure investigation of simulated coiling process: (**a**) isothermal step; (**b**) cooling to room temperature; (**c**) microstructure after cooling from 700 °C; (**d**) microstructure after cooling from 650 °C.

Figure 6 presents the dilatometric and microstructural results of the thermal simulations at 700 and 650 °C. During the isothermal step (Figure 6a), a very small increase in the relative change in length is observed. The increase was 0.014% and 0.008% at 700 and 650 °C, respectively. Such a small increase does not indicate that ferrite transformation occurred. This is support by the fact that, for both conditions, martensitic transformation detected on cooling to room temperature (see Figure 6b) is identical and close to that in Figure 1a, at ~325 °C. Consistently, the revealed microstructures (Figure 6c,d), are martensitic and similar to those also shown in Figure 5. Note that no ferrite was identified in the obtained microstructures, meaning that the selected cooling rate from 1100 down to 700 °C and then to 650 °C sufficed to avoid such transformation.

### 3.4. Intercritical Annealing 

**Figure 7:**
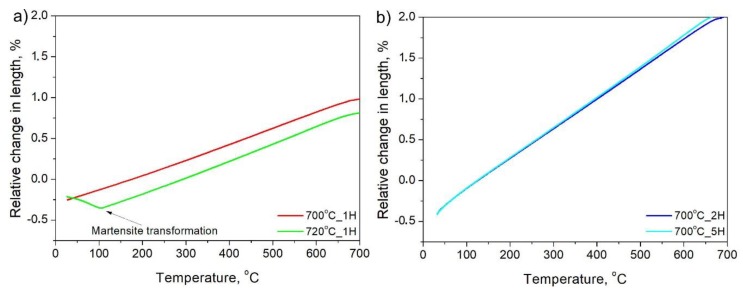
Dilatometric results during cooling to room temperature after intercritical annealing: (**a**) annealing at 700 and 720 °C for 1 h; (**b**) annealing at 700 °C for 2 and 5 h.

The third approach to obtain retained austenite in the final microstructure of the steel is intercritical annealing, variant C (Figure 1c). As already mentioned, in this variant, during heating to the selected temperatures (700–720 °C), the reverse austenite transformation from the initial martensite occurs, and the microstructure will be composed of different quantities of ferrite and austenite during annealing, which influences the subsequent phase transformation kinetics upon cooling. Therefore, this heat treatment differs from the other variants, A and B, in terms of the starting microstructure (100% austenite in variants A and B). In any case, variant C corresponds to a manufacturing schedule mostly used in case of cold-rolled steel sheets. The results of dilatometric analysis during cooling from the intercritical annealing to room temperature are presented in Figure 7. 

Differences in austenite stability are clearly detected when treating at 720 °C or a lower temperature of 700 °C. According to Figure 7a, the intercritical annealing at 720 °C is not enough to fully stabilize austenite to room temperature, and martensitic transformation is detected on cooling at 106 °C, which is much lower compared to the M_s_ measured for variants A and B, at ~325 °C. This means that some stabilization of austenite occurs during annealing. 

On the other hand, annealing at 700 °C ensures enough stability for the austenite to preserve it to room temperature. Thus, regardless of the increased annealing time from 1 to 2 and 5 h, there is no sign of martensite formation during cooling (see Figure 7a,b), which proves the high stability of austenite. It has to be highlighted that a longer time should allow manganese to diffuse between ferrite and austenite, further increasing its stability.

**Figure 8:**
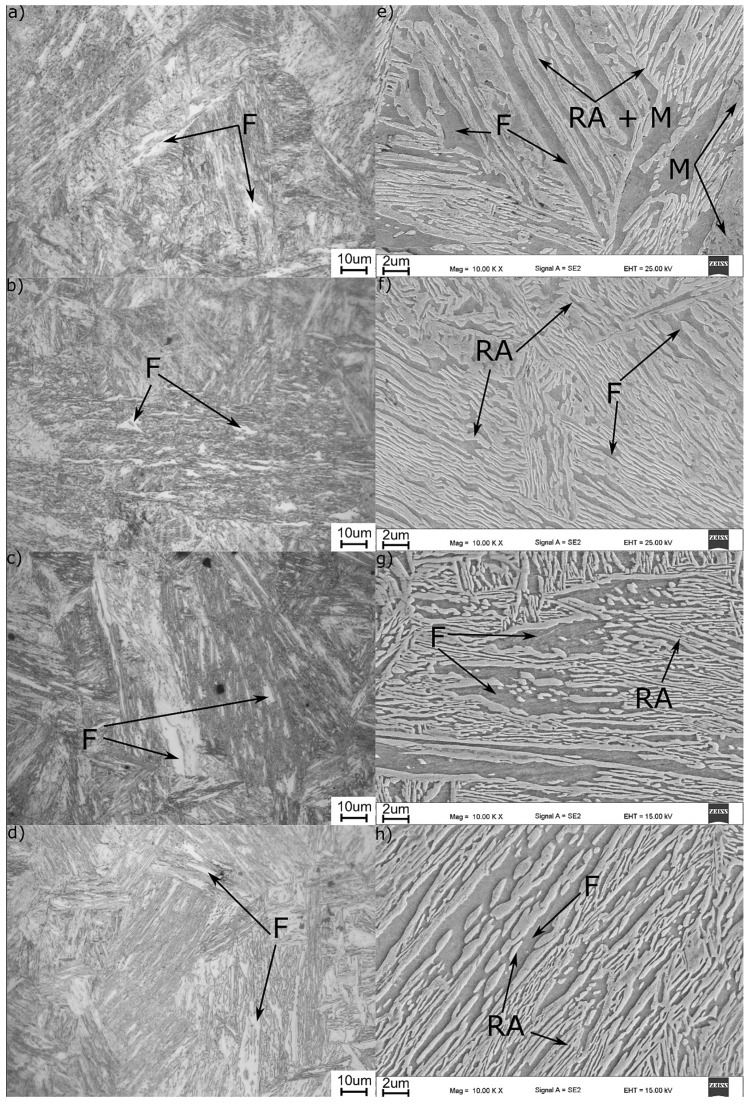
Light and scanning electron microscopy microstructures of intercritically annealed steel (**a**–**e**) 720 °C, (**b**–**f**) 700 °C, 1 h, (**c**–**g**) 700 °C, 2 h, (**d**–**h**) 700 °C, 5 h. F—ferrite, M—martensite, RA—retained austenite.

**Figure 9:**
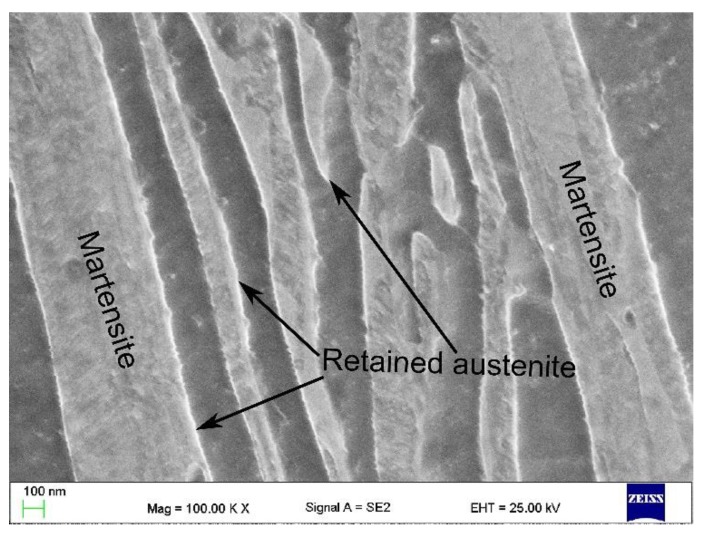
SEM micrograph of the sample intercritically annealed at 720 °C showing the retained austenite and martensite morphologies.

Figure 8 presents the LM and SEM microstructures of the analyzed annealing variants. The microstructure, in all cases, is composed of thin layers of ferrite and austenite. As the dilatometry proved, at 720 °C, some fraction of martensite can be identified. In the case of the sample intercritically annealed at 720 °C, martensite laths have a different morphology as it is presented in Figure 9. There are two morphologies: one smooth (white), which is retained austenite, and the second one, which is fragmented, corresponding to the martensite.

Prolonging the time of intercritical annealing at 700 °C leads to some growth of ferrite grains. This is shown in Figure 8c,d. However, this grain growth is lower than could be expected. The grain size is not much higher when 1, 2 and 5 h holdings are compared. Longer time influences the number of small austenite grains. For 2 and 5 h holding times, more regular austenite grains are exhibited. This may be a result of some defragmentation of austenite laths during isothermal holding.

## 4. Discussion

**Figure 10:**
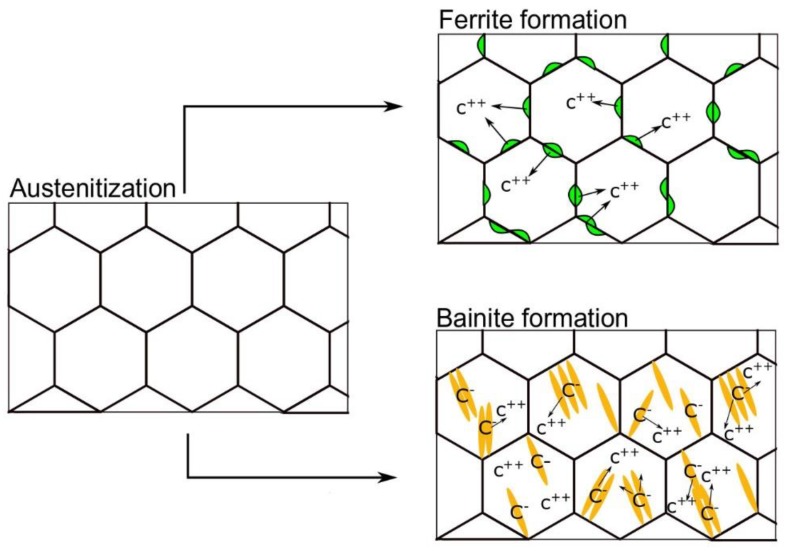
Schematic representation of carbon enrichment of the austenite during coiling and bainite transformation.

The idea of austenite stabilization during coiling simulations and isothermal bainitic transformation during cooling is presented in Figure 10, the top and down arrows, respectively. As in the case of ferrite, bainite can keep in solid solution lower amounts of carbon that in austenite. That is why, for both variants, carbon is eventually rejected from the ferritic phase to austenite. In the case of the present steel, aluminum prevents the formation of carbides in bainite. Therefore, all the carbon should diffuse to the austenite increasing its stability [11]. This process in both cases is continued to a moment when the chemical driving force for the γ/α transformation is in equilibrium. This equilibrium corresponds to the determined amount of carbon in the austenite. When this carbon amount is the same as that of equilibrium, then the transformation is stopped. However, depending on the carbon content in the austenite at equilibrium, it can be stable to room temperature or not (depending on temperature, time and chemical composition).

**Figure 11:**
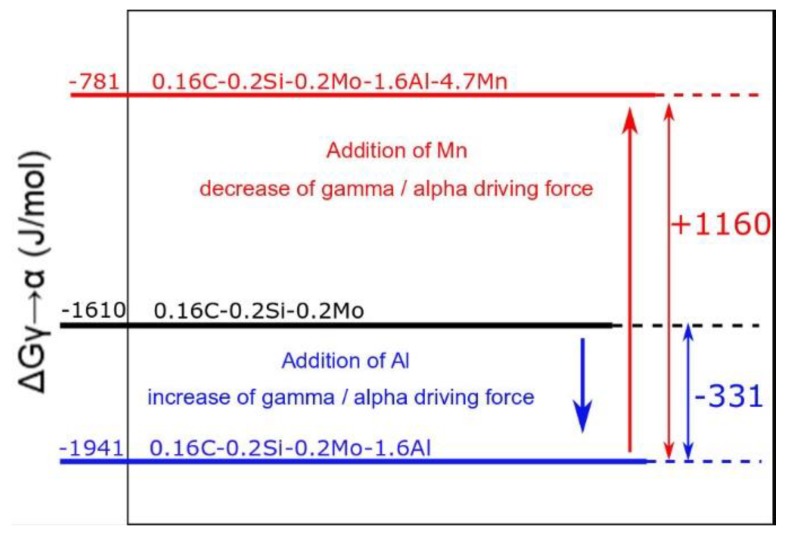
Theoretical calculations of Al and Mn effects on a driving force at 400 °C [15].

The presented results showed that the investigated steel does not exhibit any bainite or ferrite transformation. This was not expected as aluminum content at a level of 1.6% should strongly encourage the transformation, aluminum is an alpha forming element, and therefore, it is expected to accelerate bainite and ferrite reactions during heat treatment. The increase of bainitic kinetics was obtained by Tian et al. [13], who analyzed the influence of Cr and Al on the bainite transformation kinetics. The same results were obtained by Garcia-Mateo et al. [19], who stated that Al increases the driving force for gamma/alpha transformation. However, for the present steel, it was not the case. The reason for this is the high manganese content (5%), which is an austenite stabilizer. According to theoretical calculations, aluminum and manganese influence the driving force of transformation (Figure 11). However, for this steel, the effect of manganese is stronger as compared to aluminum. 

The Gibbs free energy (driving force for γ to α transformation) for pure steel (without Al and Mn) at 400 °C is −1610 J/mol. When 1.6% of aluminum is added, the driving force increases by 331 J/mol. Yet, the addition of 4.7 % of manganese decreases the driving force by 1160 J/mol. This means that, in the case of this steel, manganese is a dominant element influencing the driving force. A similar conclusion was reported by Farahani et al. [20]. Guo et al. [12] presented the results where the addition of 0.5 % manganese led to longer incubation time and a lower transformation rate. This explains why no bainitic or ferritic transformations occurred during heat treatment. Even the change in temperature, which influences the driving force, did not bring any changes [21]. Lowering the temperature increases the driving force for gamma/alpha transformation. At 350 °C, the driving force was −1034 J/mol; however, still, no transformation occurred during the isothermal holding. 

The intercritical annealing is much more appropriate treatment for this 5% Mn steel. The experimental results showed that although α’ to γ transformation occurred at both selected temperatures, 700 and 720 °C, no transformation occurred at 700 °C, as in the case for coiling simulation at the same temperature of 700 °C. Similar results were obtained by Nakada et al. [22]. They reported that the γ to α transformation did not occur even after 50 h cooling for steel containing 5% Mn. However, when they changed an approach from cooling to heating from α’ to γ (without austenitization), the transformation was present during the intercritical annealing. The reason for this was a higher austenite nucleation site density in the lath martensite and the higher Mn diffusion in α phase in comparison to the austenite. The higher stability of austenite for lower intercritical annealing temperatures corresponds to its amount, which is formed during annealing.

**Figure 12:**
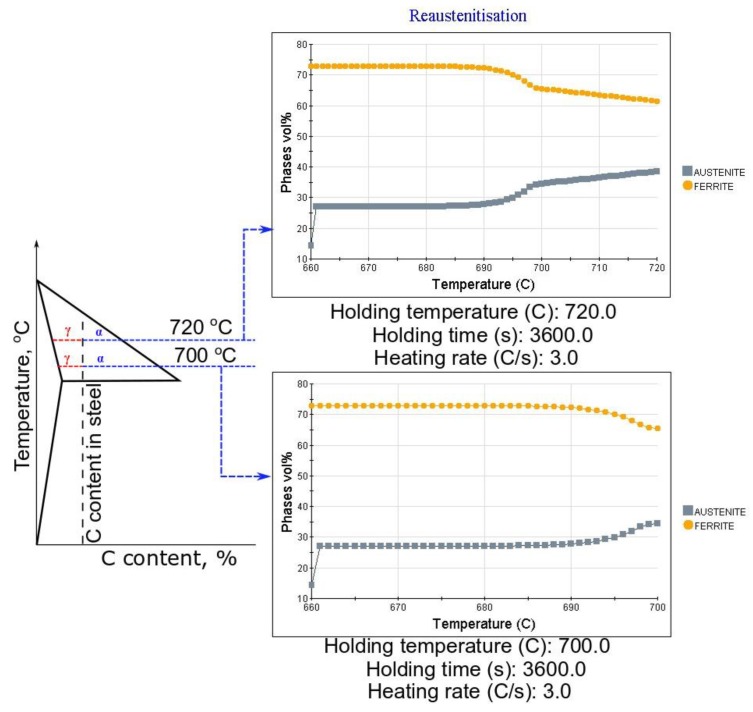
Schematic representation of a lever rule and theoretical calculations using JMatPro for intercritically annealed steels at 720 and 700 °C.

**Figure 13:**
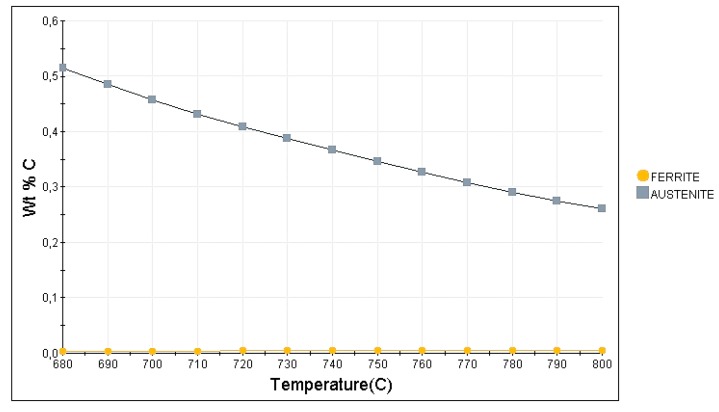
JMatPro simulation of carbon enrichment in the austenite at different intercritical temperatures.

According to JMatPro calculations in Figure 12, a higher annealing temperature leads to a higher amount of austenite in steel (40%) when compared to that at 700 °C (34%). This means that the C enrichment of austenite is lower in the former than in the later, and so it is the austenite thermal stability [23], which explains the detection of fresh martensite during cooling of samples treated at 720 °C when cooled down to room temperature. This is supported also by data in Figure 13, which shows the C content of the phases present in the microstructure as a function of the intercritical temperatures. Because ferrite rejects excess carbon to the austenite, the higher the ferrite fraction, the higher the carbon content in the newly formed austenite. According to the JMatPro calculations, such enrichment was from 0.4% to 0.46% when the temperature was decreased from 720 to 700 °C. That is why no martensite was detected upon cooling in the case of 700 °C annealing. The morphology of the final microstructure is composed of lath constituents. When the reverse austenite transformation took place, the morphology of martensite is inherited by austenite and ferrite [23,24]. Moreover, this lath morphology of austenite influences positively its stability [25,26].

## 5. Conclusions

The obtained results show that heating from the initial martensite microstructure into an interctitical region is a preferred heat treatment for the 5% Mn steel, as it is much easier to retain austenite for the intercritically processed material as compared to the case of coiling or isothermal bainitic treatments. Results showed that intercritical annealing at 720 °C allowed partial stabilization of austenite, and still, some fresh martensite was detected on cooling at the final stage. On the other hand, intercritical annealing at 700 °C allows for such carbon enrichment in the newly formed austenite that it is retained at room temperature with no sign of martensitic transformation. It has been also shown that prolonging the annealing time, for as long as 5 h, does not influence the thermal stability of retained austenite, and the only difference found with extended time is a mild effect on the size of the resulting microstructure. 

In the case of isothermal treatment and coiling simulation, no bainitic or ferritic transformations occur upon cooling from the austenitic range. This is explained by the strong effect that Mn has on reducing the chemical driving force of γ to α transformation.

## Figures and Tables

**Table 1:** Chemical composition of analyzed steel (wt.%).

C	Mn	Si	Al	Mo
0.16	4.7	0.2	1.6	0.2

## References

[1] Grajcar A., Kwasny W., Zalecki W. (2015). Microstructure-property relationships in TRIP aided medium-C bainitic steel with lamellar retained austenite. Mater. Sci. Technol..

[2] Grajcar A., Radwanski K. (2014). Microstructural comparison of the thermomechanically treated and cold deformed Nb-microalloyed TRIP steel. Mater. Technol..

[3] Smiglewicz A., Jablonska M.B. (2015). The effect of strain rate on the impact strength of the high-Mn steel. Metalurgija.

[4] Grajcar A., Skrzypczyk P., Wozniak D. (2014). Thermomechanically rolled medium-Mn steels containing retained austenite. Arch. Metall. Mater..

[5] Xie Z.J., Han G., Yu Y.S., Shang C.J., Misra R.D.K. (2019). The determining role of intercritical annealing condition on retained austenite and mechanical properties of a low carbon steel: Experimental and theoretical analysis. Mater. Charact..

[6] Zhu J., Ding R., He J., Yang Z., Zhang C., Chen H. (2017). A cyclic austenite reversion treatment for stabilizing austenite in the medium manganese steels. Scr. Mater..

[7] Chandan A.K., Bansal G.K., Kundu J. (2019). Effect of prior austenite grain size on the evolution of microstructure and mechanical properties of an intercritically annealed medium manganese steel. Mater. Sci. Eng. A.

[8] Kucerova L., Opatova K., Kana J. (2017). High versatility of niobium alloyed AHSS. Arch. Metall. Mater..

[9] Radwanski K., Kuziak R., Rozmus R. (2019). Structure and mechanical properties of dual-phase steel following heat treatment simulations reproducing a continuous annealing line. Arch. Civ. Mech..

[10] Gorka J., Opiela M. (2019). Structure and properties of high-strength low-alloy steel melted by the laser beam. Mater. Perform. Charact..

[11] Yang J.R., Huang C.Y., Hsieh W.H., Chiou C.S. (1996). Mechanical stabilization of austenite against bainitic reaction in Fe-Mn-Si-C bainitic steel. Mater. Trans..

[12] Guo H., Zhou P., Zhao A., Zhi C., Ding R., Wang J. (2017). Effects of Mn and Cr on microstructure and mechanical properties of low temperature bainitic steel. J. Iron Steel Res. Int..

[13] Tian J., Xu G., Zhou M., Hu H., Wan X. (2017). The effects of Cr and Al addition on transformation and properties in low-carbon bainitic steels. Metals.

[14] Sente Software Ltd (2005). A Collection of Free Downloadable Papers on the Development and Application of JmatPro. http://www.sentesoftware.co.uk/biblio.html.

[15] Bhadeshia H.K.D.H. (1982). A thermodynamic analysis of isothermal transformation diagrams. Metal Sci..

[16] ASTM A1033-04 (2004). Standard Practice for Quantitative Measurement and Reporting of Hypoeutectoid Carbon and Low-Alloy Steel Phase Transformations.

[17] Klueh R.L., Maziasz P.J., Lee E.H. (1988). Manganese as an austenite stabilizer in Fe-Cr-Mn-C steels. Mater. Sci. Eng. A.

[18] Navarro-Lopez A., Sietsma J., Santofimia M.J. (2016). Effect of prior a thermal martensite on the isothermal transformation kinetic below M_s_ in a low-C high-Si steel. Metall. Mater. Trans. A.

[19] Garcia-Mateo C., Caballero F.C., Bhadeshia H.K.D.H. (2003). Acceleration of low-temperature bainite. ISIJ Int..

[20] Farahani H., Xu W., Zwaag S. (2018). Predicting the cooperative effect of Mn-Si and Mn-Mo on the incomplete bainite formation in quaternary Fe-C alloys. Philos. Mag. Lett..

[21] Shah M., Das S.K., Chowdhury S.G. (2019). Effect of alloying elements on microstructure and mechanical properties of air-cooled bainitic steel. Metall. Mater. Trans. A.

[22] Nakada N., Mizutani K., Tsuchiyama T., Takaki S. (2014). Difference in transformation behavior between ferrite and austenite formation in medium manganese steel. Acta Mater..

[23] Yunbo X., Zou Y., Hu Z., Han D., Chen S., Misra R. (2017). Correlation between deformation behavior and austenite characteristics in a Mn-Al type TRIP steel. Mater. Sci. Eng. A.

[24] Shu Y., Liu X., Liang T., Zhao Y. (2018). The effects of the initial microstructure on microstructural evolution, mechanical properties and reversed austenite stability of intercritically annealed Fe-6.1Mn-1.5Si-0.12C steel. Mater. Sci. Eng. A.

[25] Xiong X.C., Chen B., Huang M.X., Wang J.F., Wang L. (2013). The effect of morphology on the stability of retained austenite in quenched and partitioned steel. Scripta Mater..

[26] Steineder K., Schneider R., Krizan D., Beal C., Sommitsch C. (2015). Comparative investigation of phase transformation behavior as a function of annealing temperature and cooling rate of two medium-Mn steels. Steel Res. Int..

